# Machine learning in the prediction of human wellbeing

**DOI:** 10.1038/s41598-024-84137-1

**Published:** 2025-01-10

**Authors:** Ekaterina Oparina, Caspar Kaiser, Niccolò Gentile, Alexandre Tkatchenko, Andrew E. Clark, Jan-Emmanuel De Neve, Conchita D’Ambrosio

**Affiliations:** 1 London School of Economics, London, UK; 2 Warwick Business School, Coventry, UK; 3 University of Oxford, Oxford, UK; 4 University of Luxembourg, Esch-sur-Alzette, Luxembourg; 5 PSE-CNRS, Paris, France

**Keywords:** Subjective wellbeing, Prediction methods, Machine learning, Psychology, Human behaviour

## Abstract

Subjective wellbeing data are increasingly used across the social sciences. Yet, despite the widespread use of such data, the predictive power of approaches commonly used to model wellbeing is only limited. In response, we here use tree-based Machine Learning (ML) algorithms to provide a better understanding of respondents’ self-reported wellbeing. We analyse representative samples of more than one million respondents from Germany, the UK, and the United States, using data from 2010 to 2018. We make three contributions. First, we show that ML algorithms can indeed yield better predictive performance than standard approaches, and establish an upper bound on the predictability of wellbeing scores with survey data. Second, we use ML to identify the key drivers of evaluative wellbeing. We show that the variables emphasised in the earlier intuition- and theory-based literature also appear in ML analyses. Third, we illustrate how ML can be used to make a judgement about functional forms, including the existence of satiation points in the effects of income and the U-shaped relationship between age and wellbeing.

## Introduction

A substantial and interdisciplinary literature on the correlates and determinants of subjective wellbeing has emerged over the past 50 years^1^. In parallel, international organisations^2^ and national governments^3^ have turned to subjective wellbeing data as a key tool for policy analysis.

Subjective wellbeing data has been extensively validated^4–6^, and has been shown to correlate well with objective outcomes^7^. Generally, such data also do better in predicting future behaviour than many other standard social science variables^8–10^. On that basis, we might expect that the answers respondents provide to questions about their wellbeing are well-predicted using standard regression equations. However, to the contrary, and despite the widespread use of these scores, our current ability to model wellbeing is surprisingly limited. Standard approaches, where variables are selected based on intuition or theory, explain relatively little of the variation. Individual-level models typically yield R-squared figures of no more than 15% (Ref.^11^ is one typical example). Yet, especially in economics and psychology, the prediction and explanation of individual wellbeing are one of the discipline’s core tasks. Our limited ability to make predictions would thus seem to be a major shortcoming.

This notwithstanding, the existing literature has largely reached a consensus on the main correlates of wellbeing. These include good health^12,13^, unemployment^14,15^, social relations^16,17^, as well as personality traits^18–20^. For wider overviews of the interdisciplinary literature, see^21–25^.

Nevertheless, some questions are still actively debated. Two examples are whether wellbeing is U-shaped in age (e.g. Ref.^26^ versus Refs.^27,28^), and whether income beyond a ‘satiation point’ yields no further increase in wellbeing (e.g. Refs.^29,30^ versus Refs.^31,32^). The answers researchers provide to these questions depend on their prior beliefs and their subsequent modelling choices. Machine learning (ML) algorithms, on the contrary, are indifferent about the conclusions they reach: they have no ‘axe to grind’. The use of ML would therefore seem to be particularly apt for the resolution of controversial academic debates in a disinterested manner. We therefore investigate the effects of income and age on subjective wellbeing using this approach.

We pose three research questions:

- **RQ1:** Do ML algorithms predict wellbeing substantially better than conventional linear models, and what is the upper limit on our ability to predict wellbeing based on survey data?
- **RQ2:** Are the variables that ML algorithms identify as important in the prediction of wellbeing aligned with those commonly emphasised in the literature?
- **RQ3:** Can ML help to resolve ongoing debates about the specific shape of the relationships between wellbeing and key socio-economic variables?

To our knowledge, this paper represents the first systematic attempt to evaluate the (dis-) advantages of using ML for the analysis of wellbeing at a global scale. Earlier work focused on single drivers of wellbeing like age^33^ and relatively limited country-, year- or age-specific samples^34,35^, or used objective biomarkers in small-N studies^36^.

We apply random forests^37,38^, gradient boosting^39,40^, and penalised regressions^41^. Random forests and gradient boosting are tree-based algorithms that have been shown to perform well with standard social-science data that is organised in rows and columns (i.e. ‘tabular data’, as opposed to text or images: see^42^). Penalised regressions are a convenient tool for analyses that involve a large number of covariates, as will be the case in some of our specifications^41^. In preliminary analyses, we also evaluated the use of feed-forward neural networks. Since they typically performed no better than Ordinary Least Squares (OLS), we did not consider them further (c.f.^43^).

We focus on life satisfaction as a key cognitive-evaluative measure of wellbeing^44^. In supplementary analyses, we also analyse positive affect (as measured by e.g. the rate of smiling) and negative affect (as measured by e.g. the rate of feeling anger). Our main findings extend to these measures (see Online Appendix A1). Our analyses are based on three of the largest currently-available datasets including regular information on wellbeing: the German Socio-Economic Panel (SOEP), the UK Household Longitudinal Study (UKHLS) and the American Gallup Daily Poll. See the online methods section on details regarding our data and empirical approach.

As is standard in the ML literature—but not so in psychology and the social sciences—we exclusively evaluate model performance using data that these models have not previously seen (the ‘test’ set). Model parameters are determined via a separate ‘training’ set. Model evaluation then refers to the quality of the out-of-sample prediction, as the test and training sets do not overlap. Importantly, there is no automatic improvement in model performance by the addition of more variables: any advantage of ML models over OLS cannot therefore be attributed to mechanical overfitting.

The performance of ML methods can be used to benchmark the highest possible predictive ability provided by a given set of characteristics (see^45^). Regarding **RQ1**, we find that ML algorithms predict better than standard linear models. The size of this improvement is moderate in absolute terms, but substantial when compared to the predictive power of key variables such as health.

That said, much greater improvements come from adding individual characteristics to the model. Model performance, as judged on the unseen ‘test set’ data, roughly doubles for both the OLS and ML approaches when the set of variables is expanded from a standard set (we call this the ‘Restricted Set’) to all of the available data (the ‘Extended Set’). Independently of the type of algorithm, an R-squared of around 0.30 appears to be the feasible maximum for individual-level models in survey data. This is approximately half of all the predictable wellbeing variance, as determined by the test–retest correlations obtained in earlier work^46^.

For **RQ2**, our data-driven ML results align with the conventional literature. Variables reflecting respondents’ social connections, health, and material conditions are consistently the most predictive of their wellbeing. The extended-set analysis identifies the following additional variables as being particularly important in predicting self-assessed wellbeing scores: personality traits, relationship quality, additional measures of health, and perceptions of the local area. Many of these are not consistently included in wellbeing analyses. There is a substantial correlation in variable-importance rankings across algorithms ($\rho =0.58$ to $\rho =0.83$). Hence, ML approaches and OLS agree on what matters for wellbeing.

Last, with respect to **RQ3**, we find support for a U-shaped relationship between age and wellbeing in all three datasets. For income, there is strong evidence of satiation for equivalent household incomes of over 40,000 GBP (50,000 EUR) in the UK (German) data. Yet, we do not find evidence of satiation in the US.

## Results

We use the data from three nationally-representative surveys: the German Socio-Economic Panel (SOEP), the UK Longitudinal Household Survey (UKHLS) and the US Gallup Daily Poll (Gallup). These are three of the most widely-used datasets in the Social Sciences. For each dataset, the results are presented for models with two sets of explanatory variables: a **restricted set** and an **extended set**.

Our selection of the variables included in the **restricted set** is informed by those commonly used in prior research, such as^47^ or^16^. This set includes age, sex, health status, household composition, along with various socioeconomic and demographic factors. This strategy enables a comparative analysis of ML algorithms against OLS in a conventional estimation context. Furthermore, we have chosen variables to enable substantial overlap across datasets, allowing for comparisons of findings across three countries.

Our **extended sets** include all variables present in each survey, excluding those that directly measure subjective wellbeing (e.g., domain satisfaction, happiness, subjective health) and mental health, along with meta-data like respondent identification numbers. This approach leverages the strength of machine learning algorithms in handling a large number of explanatory variables. However, due to variation in the set of available variables across surveys, the findings for different countries may not be directly comparable.

We rely on four estimation approaches. We start with Ordinary Least Squares (OLS) regressions, which is the standard approach in the literature. We employ Least Absolute Shrinkage and Selection Operator (LASSO) as a device for variable selection. We then consider two regression tree-based ML algorithms - Random Forests (RF) and Gradient Boosting (GB).

The details on the data sources, the included variables and the algorithms can be found in the Methods Section.

### Model performance

We begin with RQ1: whether ML algorithms substantially outperform OLS (the most-common approach in the existing literature) in predicting wellbeing.

#### The restricted set of explanatory variables

In this section we start by investigating the performance of our models using a ‘restricted’ set of covariates.

**Fig. 1:**
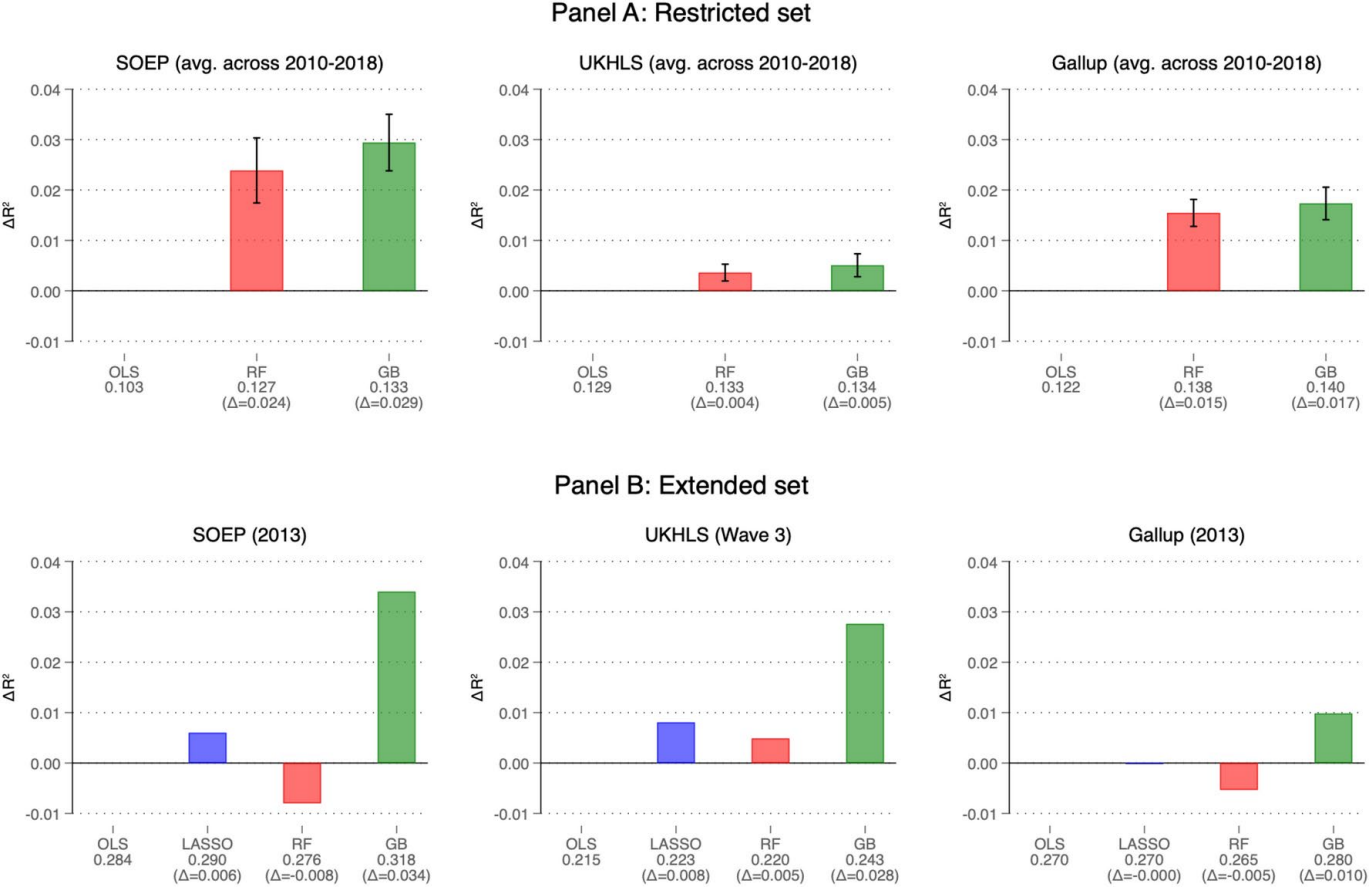
Differences in out-of-sample performance between OLS and ML. Panel (**A**) shows the differences in the mean R-squared figures between OLS and gradient boosting (GB)/random forest (RF), using the restricted set of variables. The bars refer to the average of the separate estimates for each of the years (2010–2018), and the whiskers show the standard deviations of these yearly estimates. Panel (**B**) shows the analogous figures for the extended set of variables, comparing OLS with LASSO, GB and RF. 2013 data are used here. All R-squareds are calculated from the unseen test data. The figures below each bar indicate the absolute out-of-sample R-squared and those in parentheses the differences in the out-of-sample R-squared compared to OLS. Throughout, gradient boosting (GB) yields the best predictive performance.

Panel A of Fig. 1 depicts the improvements in each algorithm’s performance over OLS. The results here are based on the ‘test-set’ (see Appendix Figure A2 for results based on the training set), and can thus be interpreted as an assessment of the models’ ability to make out-of-sample predictions. We use the R-squared as our primary evaluation metric, to facilitate comparisons with previous analyses.

In Panel A of Fig. 1 each algorithm is trained separately for each year between 2010 and 2018. For each year we take the difference between the R-squared from each ML algorithm and the R-squared from the OLS estimation. The figure reports the average differences across these years. In absolute terms, the R-squareds are very similar across datasets, ranging from 0.10 (SOEP) to 0.13 (UKHLS). Gradient boosting (GB) and random forests (RF) yield larger R-squared values than OLS in each case. Specifically, random forests yield increases in R-squared of 0.024 (SOEP), 0.004 (UKHLS) and 0.015 (Gallup); the respective improvements from using gradient boosting are slightly larger at 0.029, 0.005, and 0.017. ML algorithms thus do outperform linear regressions, and gradient boosting always outperforms random forests. Although our main focus is here on cross-sections, we show in online Appendix A.2 that substantially the same results are obtained when exploiting longitudinal panel information.

On their own, these gain figures are hard to interpret. We therefore illustrate their size by comparing them to the change in predictive performance when omitting the respondent’s health status—a key wellbeing predictor—from the baseline OLS regressions. These results are shown in Table 1. The first two columns in Panel A of the table list the OLS test-set R-squared figures with and without health. Column 3 shows the R-squared from gradient boosting. Benchmarking one against the other, the improvement in prediction from gradient boosting (our best ML algorithm) is between 15 and 104% of the role of health in predicting wellbeing. Evaluated in this way, the gains from using ML are not negligible.

**Table 1:** An illustration of the performance improvement from using ML.

	OLS, full	OLS, no health	GB	GB gain as % of loss from removing health
Panel A: Restricted set of variables				
SOEP	0.103	0.075 ($\Delta =-0.028$)	0.133 ($\Delta =0.029$)	104%
UKHLS	0.129	0.095 ($\Delta =-0.034$)	0.133 ($\Delta =0.005$)	15%
Gallup	0.122	0.093 ($\Delta =-0.029$)	0.140 ($\Delta =0.017$)	59%
Panel B: Extended set of variables				
SOEP	0.284	0.240 ($\Delta =-0.043$)	0.318 ($\Delta =0.034$)	79%
UKHLS	0.215	0.197 ($\Delta =-0.018$)	0.243 ($\Delta =0.028$)	156%
Gallup	0.270	0.240 ($\Delta =-0.031$)	0.280 ($\Delta =0.010$)	32%

The figures refer to the R-squared values from the test set. Figures in parentheses show differences in R-squared compared to the full OLS specification. In the extended specifications (Panel B), multiple health variables are dropped in each dataset. Specifically, we drop 21, 19 and 12 health-related variables in the Gallup, the SOEP and the UKHLS respectively

#### The extended set of explanatory variables

Vastly expanding the set of covariates beyond what is standard will (weakly) increase our ability to explain wellbeing in the training set. It is also possible, although far from mechanical, that they will improve our ability to predict wellbeing in the test set. Given the greater flexibility of the ML algorithms, we may expect these latter to benefit more from additional variables than OLS. The extended sets of variables we consider here include all of the variables available in the 2013 waves of the SOEP and Gallup, and Wave 3 (covering 2012–2014) of the UKHLS. See the Methods Section for further details.

Panel B of Fig. 1 depicts the results using this extended set of variables. The R-squared for the extended set of variables is around double that for the restricted set in all of the algorithms. The OLS R-squared is now 0.28 for the SOEP, 0.22 in the UKHLS, and 0.27 for Gallup. Thus, the kinds of standard specifications often used in the field, and which were used in the previous section, do not fully exploit the predictive information available in typical survey data. It is also worth re-emphasising here that these R-squared figures are based on evaluating models on the test set. They therefore do not reflect merely mechanical increases in the share of explained variance in the training set due to adding more variables to the model. Results using the training set are given in Panel B of Online Appendix Figure A.2.

Given the large number of variables in the extended set, we now also estimate LASSO regressions, which serve as a device for variable selection (see our Methods Section). LASSO regressions marginally outperform the corresponding OLS models. Gradient boosting remains the best-performing algorithm and clearly predicts better than OLS. The absolute gain in the R-squared from gradient boosting over OLS is now 0.034, 0.028 and 0.010 for the SOEP, UKHLS and Gallup respectively. Random forests now perform poorly, and worse than OLS for SOEP and Gallup. This has also been observed in other empirical applications where covariates were measured with error^48^.

We again compare the performance gains from gradient boosting to those from the inclusion of health information in OLS estimations. As shown in Panel B of Table 1, the results again indicate that the gains from using gradient boosting are similar to the role of health in predicting wellbeing.

We thus conclude that tree-based ML algorithms can predict wellbeing better than conventional methods. These gains are moderate in absolute terms, but more substantial when compared to the predictive power of health. However, these gains do come from algorithms that take roughly 100 times longer to estimate. ML algorithms thus involve a trade-off between computational burden and predictive performance.

### Variable importance

**Fig. 2:**
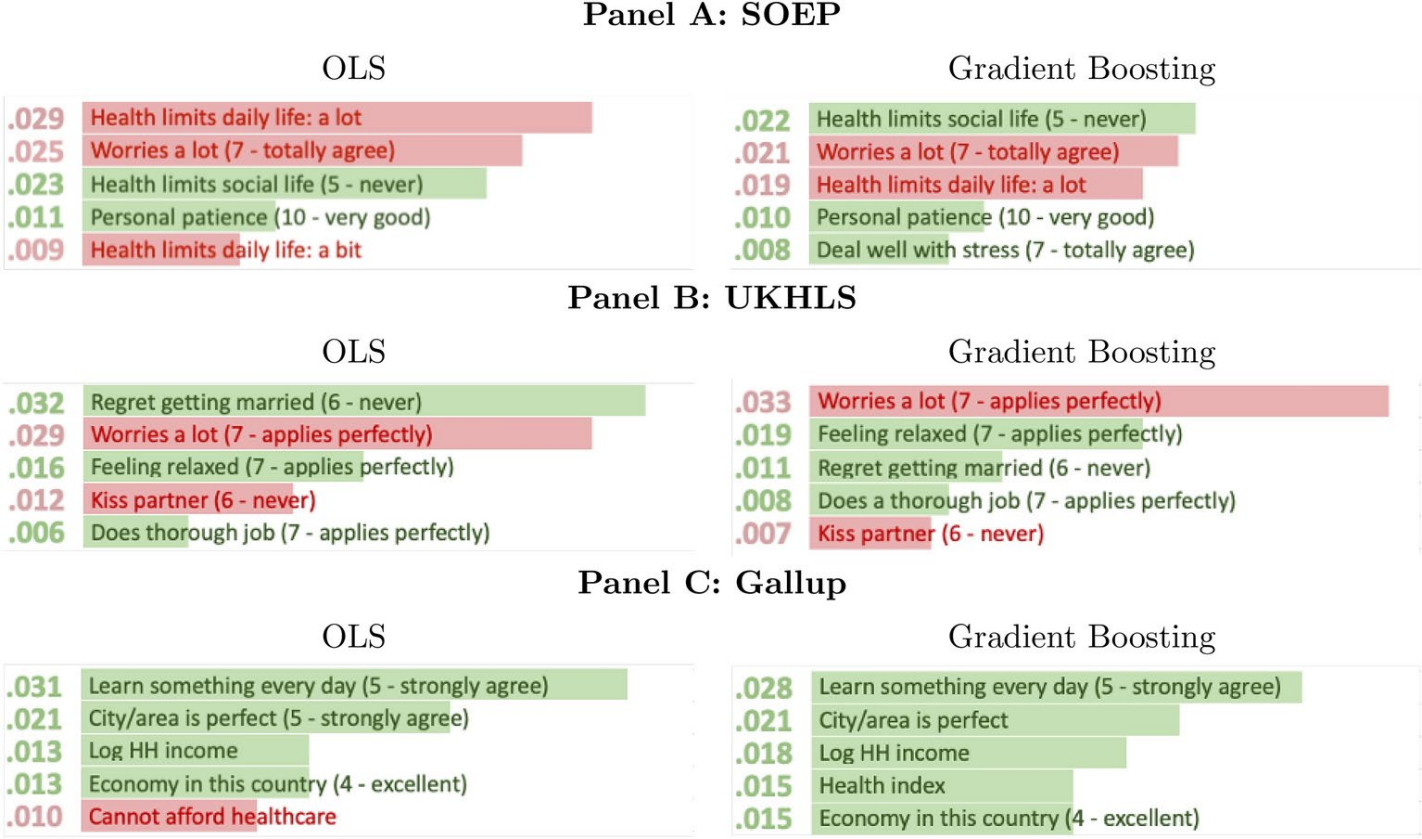
Permutation importance and pseudo partial effects of OLS and GB on the extended set of variables: the five most-important variables. The bars and numerical values represent permutation importances. They are coloured red for variables with negative pseudo partial effects and green otherwise. For Likert-scale variables, the highest category is reported.

We now turn to RQ2: based on estimating permutation importances across the extended set of variables, we assess whether the variables that ML identifies as important in predicting life satisfaction align with those from the conventional literature. Figure 2 lists the five most-important variables identified in OLS and GB (the best-performing ML algorithm) in each dataset. Online Appendix Table A4 further shows extended results for the Top-10 most-important variables for OLS, RF and GB. The bars and numerical values refer to permutation importance, i.e. the drop in the R-squared when the values of the variable are randomly permuted across respondents. The variables that are negatively associated with average wellbeing are in red, and those with a positive association in green. In each country, individual health and interpersonal relationships are among the most-important predictors. As expected, respondents whose health limits their activities are on average less satisfied with their lives, while people with fulfilling relationships are typically more satisfied. The directions of the estimated effects are in line with those in previous conventional work. ML algorithms and OLS thus generally agree on the direction and approximate size of the most-important variables (see Online Appendix Table A4 for the effect-size estimates).

A more-systematic measure of agreement between ML and OLS is given by the rank correlations (in terms of their permutation importance) of each variable across algorithms and datasets. The results in Online Appendix Table A5 reveal strong agreement between GB and RF, with the rank correlation figure never being below 0.79. The correlations with the OLS ranking are somewhat lower, with a minimum value of 0.58. Nevertheless, we can strongly reject ($p<0.001$) the null hypothesis that the rankings are uncorrelated, supporting our conclusion that the OLS and ML algorithms are in broad agreement.

Apart from the conventional variables used in wellbeing analyses, such as health and interpersonal relationships, personality traits are also identified as being important in the UKHLS and SOEP (personality traits are not measured in the Gallup survey). This is in line with earlier work showing strong links between personality and wellbeing^49,50^. In the UK, worrying a great deal and feeling very relaxed appear in the top-three wellbeing predictors. In the German data, worrying and patience are in the top-four predictors in both OLS and Gradient Boosting.

Beyond these similarities, there are some cross-country differences. The most striking concerns financial factors. These are important in the US (e.g., household income and being able to pay for healthcare) but not in the other countries. To see whether this is a genuine finding or due to the extended-set variables being different across countries, we reproduce the analysis for the restricted set, which has a strongly overlapping set of variables. There, the cross-country differences in the importance of income largely disappear. More generally, most of the variables identified as important in these harmonised datasets are very similar across the three countries (see Online Appendix Table A6). They include health, income, marital and employment status, as well as home-ownership—a proxy for wealth—and age. However, sex and ethnicity are only important in the US. Education is among the most important factors in the US and Germany, but not in the UK.

It is of interest to reflect upon these remaining country differences. In terms of sex and ethnicity, the US has a worse Gender Inequality Index ranking than either the UK or Germany in all of the years 1990–2022. More generally, in the index of equal treatment and absence of discrimination produced by the World Justice Project, Germany ranks 9th, the UK 25th, and the USA 103rd (out of 140 countries). Regarding the lesser importance of education in the UK, OECD research has shown that the earnings reward from education is relatively low in the UK: out of 37 countries, the US ranked 3rd in this respect, Germany 9th, and the UK 27th^51^.

### Wellbeing by age and income

Finally, we turn to RQ3. Whether the relationship between age and wellbeing is U-shaped, and whether there is a satiation point beyond which income no longer yields wellbeing are two open and hotly-debated questions. Tree-based algorithms freely estimate the most-appropriate functional forms. They are thus particularly well-suited to act as agnostic judges in these debates.

**Fig. 3:**
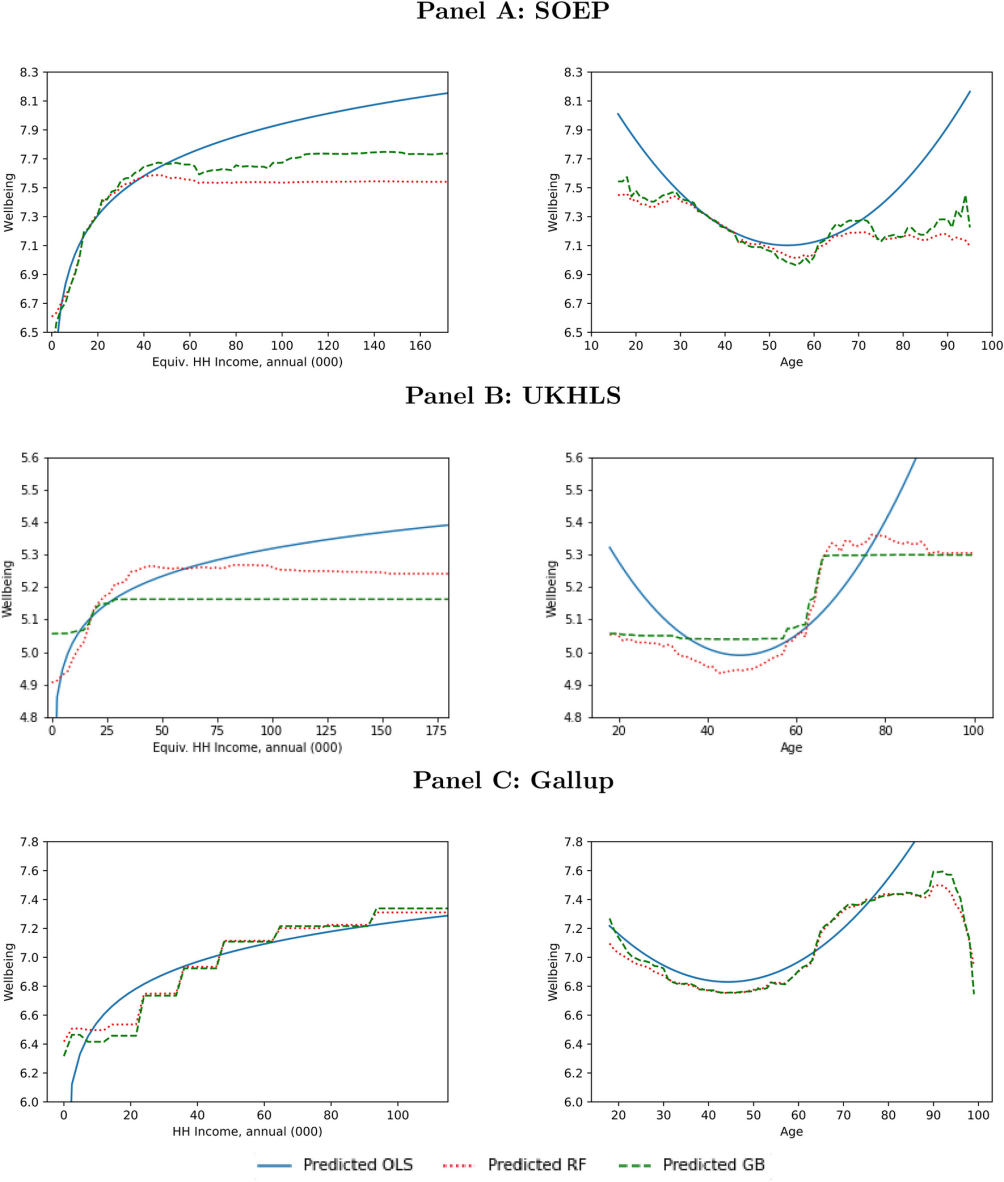
Relationships between wellbeing, age, and household income; conditional on the other variables in the restricted set. Income is continuous in the SOEP and the UKHLS, and we use equivalence-scale adjusted household income in the analysis. For ease of presentation, we only depict the relationship up to equivalent household income figures of 180,000 in the local currency for these two datasets. Income is collected in income bands in Gallup, and there is no information on household size in 2013. The Gallup analysis thus refers to unadjusted household income.

Figure 3 and Online Appendix Figure A3 depict average predicted wellbeing for different levels of age and income, holding the other covariates constant. In the OLS estimation, illustrated in blue, we assume a quadratic functional form for age and a log-linear functional form for income: these are both extremely common in the existing literature. The relationships estimated using RF are plotted in red, and those using GB in green.

Both ML algorithms track the log-linear functional form for income over much of the income distribution. However, once we reach relatively-high equivalised annual incomes of 50,000 EUR in the SOEP and 40,000 GBP in the UKHLS, ML suggests that wellbeing no longer rises with income. We cannot confirm this finding in the Gallup data, where income is measured in discrete bands with a highest value of 100,000 USD or above (equivalent to 70,000 GBP or 78,000 EUR in 2013; Since income data in the US is collected in bands, the algorithms model the relationship between income and wellbeing as a step function. Of course, this result is unlikely to also hold were the US income information to be recorded as a continuous variable, as is the case in the UK and German datasets.) As we also cannot correct for household size, income in Gallup is not comparable to the adjusted equivalent incomes in the SOEP and UKHLS. Given these caveats, and in line with previous work on wellbeing from the US^29,32^, we find no evidence of satiation in the relationship between income and evaluative wellbeing in the Gallup data.

With respect to the relationship between age and wellbeing, both ML estimations replicate the well-known U-shape up to age 70. This pattern appears in all countries, and is the least-pronounced in Germany and the most-pronounced in the US. However, unlike the smooth quadratic U-shape in the OLS results, we find a much more pronounced ‘kink’ at around age 65 in ML, which we suspect reflects higher wellbeing around the age of retirement^52,53^. Moreover, and in particular for the US, there is a steep drop in predicted wellbeing above age 90. Our results are then in line with the parametric findings in Ref.^27^ of a clear U-shape in wellbeing during working age. They are also in line with the neural network-based results of^33^, which focused on Germany only.

## Discussion

We draw four main conclusions.

First, tree-based ML approaches do indeed perform better at predicting wellbeing than conventional linear models. Gradient boosting consistently outperforms random forests. Although the gains in R-squared are modest in absolute terms, they are comparable with—and sometimes exceed—the extent to which information on respondents’ health adds to wellbeing predictions. This finding is not mechanical: Performance is evaluated out of sample so that there is no guarantee that an unconstrained functional form will perform better^54,55^. The improved performance rather implies that there are genuine non-linearities in the drivers of wellbeing.

Second, when we use all of the non-wellbeing variables available as predictors, we more than double the explained variation in wellbeing for all estimation methods. The R-squared figure with this extended set of variables is around 0.3, which looks to be the maximum achievable with the current survey data. This is approximately half of the predictable wellbeing variance, as defined by test-retest correlations found in earlier work^46^. Hence it seems that even if we use all the information available in standard social surveys, we still fail to explain about half of the in-principle explainable variance in individual wellbeing.

Third, almost all of the variables that turn out to be important in the extended-set specifications relate to health, economic conditions, personality traits, and personal relationships. This purely data-driven process thus picks out the same core determinants of wellbeing as have been identified in the earlier conventional literature^1^. In that sense, machine-learning approaches validate the previous human-guided search for the determinants of wellbeing. We believe that ML techniques are exceptionally well-equipped for this validation exercise. This looks to be good news for the field.

Unlike OLS, where functional forms are imposed between the covariates and the outcome, random forests and gradient boosting involve no such strong a priori assumptions. We have here considered the relationships between wellbeing, income and age. Our last finding is that this data-driven approach—which does not require the researcher to decide on the functional form ex ante—provides support for the U-shape in age and, where comparable data is available, a satiation point beyond which higher incomes are unrelated to wellbeing.

We see two directions for future research. The first is to further explore the capabilities of ML models, e.g., by combining unsupervised and supervised learning. For example, unsupervised techniques can split the data into overlapping clusters of individuals, where the predictive advantage of supervised non-linear ML models may be more effectively used within such clusters. Moreover, the analysis here has been correlational, identifying key variables for the prediction of wellbeing. A natural next step is to apply machine learning to the variables that matter most for wellbeing in a causal sense^56^.

The second direction is to extend this analysis beyond rich Western countries. A key question is whether our findings replicate in a more global setting. Insofar as the scope for improving wellbeing is greater in low- and middle-income countries^57–59^ applying ML approaches in this setting may be particularly valuable going forward.

## Methods

In this section we describe in more detail the methods used throughout the manuscript. Our methods were carried out in accordance with all relevant guidelines and regulations. Since this study relied on secondary observational data, formal ethical approval was not sought for this study.

### Algorithms

We model wellbeing using four kinds of algorithms.

First, as our baseline and corresponding to the workhorse of a great deal of research on subjective wellbeing, we estimate **Ordinary Least Squares (OLS)** regressions. When using OLS, the researcher implicitly assumes that reported wellbeing is a linear combination of the chosen set of explanatory variables. OLS estimates can have large variances when the number of explanatory variables is large, leading to poor predictions.

The second algorithm, the **Least Absolute Shrinkage and Selection Operator (LASSO)**, tackles this issue by adding a penalty for the sum of squared coefficient magnitudes. The LASSO tends to shrink coefficients on variables with little explanatory power to zero. In some specifications, we thus use LASSO as a device for variable selection.

The third and fourth algorithms we consider—**Random Forests (RF)** and **Gradient Boosting (GB)**—are based on regression trees^60^. Specifically, **Random Forests**, the third algorithm we consider, average across a large number of trees (set to 1000 throughout). Each individual tree is grown on a separate bootstrap sample of the original data. At each split, only a random subset of all covariates is considered. Both operations reduce the correlation between trees, thereby reducing the variance of the resulting overall predictions. The fourth algorithm, **Gradient Boosting**, proceeds by sequentially fitting regression trees on the residuals of the predictions of the previous collection of trees. Intuitively, each subsequent tree attempts to explain the variance that was not explained by the previous trees.

The algorithms are trained on the training set, which here contains 80% of the sample. Each algorithm’s performance is on the contrary evaluated only with the unseen test set, which contains the remaining 20% of observations. The optimal hyperparameters are chosen via 4-fold cross validation on the training-set data. The chosen hyperparameters can be found in Online Appendix Table A1. We evaluate the stability of our results over time, where feasible, by training each algorithm separately on each wave within each survey. All algorithms are implemented with the scikit-learn (v1.2.2) library in Python^61^. Please see Appendix A.3 for a more detailed description of estimations methods.

Linear models are a standard approach in the wellbeing literature (see e.g. the papers reviewed in^23^). While traditional statistical methods are capable of modelling nonlinear relationships—indeed, standard wellbeing models often enter the natural logarithm of income and employ polynomials for age to reflect a U-shaped wellbeing-age relationship—these modelling choices are predetermined by the researcher, guided by intuition and prior knowledge. In contrast, the ML techniques we use allow for the selection of functional forms to be driven by the data itself, rather than requiring strong a priori decisions by the researcher.

### Data

We analyse the data from three nationally-representative surveys over the 2010 to 2018 period: the German Socio-Economic Panel (SOEP), the UK Longitudinal Household Survey (UKHLS) and the US Gallup Daily Poll (Gallup).

The SOEP and UKHLS are accessible for academic researchers. The Gallup data is not publicly accessible. Data access for this project was granted on the basis of Jan-Emmanuel De Neve’s status as research advisor to Gallup. See the data availability statement for further details on how to gain access to these data.

The Gallup data covers the US adult population, with daily cross-sectional telephone-based surveys (annual N = 115,192 to N = 351,875 after removing incomplete information). Self-reported evaluative wellbeing is measured by the Cantril Ladder of Life^62^, which is recorded on a scale from 0 to 10. The SOEP and UKHLS are respectively representative of the German and UK adult population, with interviews conducted in person^63,64^. To enable comparison with the Gallup data, we consider the survey period between 2010 and 2018 (SOEP annual N = 26,089 to N = 32,333; UKHLS annual N = 29,605 to N = 40,679). Life satisfaction is measured on a scale from 0 to 10 (SOEP) or 1 to 7 (UKHLS). The descriptive statistics and histograms of each wellbeing measure appear in Online Appendix Figure A1. For each of the datasets we used, participants gave informed verbal consent to participate in the survey.

### Explanatory variables

We evaluate each algorithm’s performance for two different sets of explanatory variables.

We first consider a **restricted set** of variables that are similar in all three datasets. This set includes: sex, age, age-squared, ethnicity, religiosity, number of household members, number of children in the household, marital status, log household income (equivalised used the modified OECD scale), general health status, disability, body mass index (BMI), labour-force status, working hours, home ownership, area of residence, and interview month. A more-detailed description of these variables appears in Online Appendix Table A2.

We second consider much-larger **extended sets** of explanatory variables. Here, we only use the 2013 wave of Gallup and SOEP, and Wave 3 of the UKHLS (which covers 2011–2012). These waves/years were chosen as they include personality traits in the SOEP and UKHLS. Our extended sets include all of the available variables in each survey, apart from variables that are direct measures of subjective wellbeing (such as domain satisfaction, happiness and subjective health) and mental health, as well as extraneous meta-data (such as respondents’ identification numbers). The resulting Gallup dataset contains 67 variables, and around 450 variables are retained in the SOEP and UKHLS. The variables concern the respondents’ family relationships, social life, neighbourhood and residence, incomes and expenditures, attitudes, personality traits and other characteristics. The summaries of the variables in each dataset are presented in Online Appendix Table A3. We exclude variables with more than 50% missing values. Missing values for continuous variables are assigned the observed means, while missing values for categorical variables are assigned a new category. We convert categorical variables into sets of dummy variables, one for each category. Creating these dummies and removing perfectly collinear variables yields 210, 542, and 957 effective explanatory variables in the Gallup, SOEP and UKHLS datasets respectively.

Some variables have no predictive power. We therefore use LASSO as a device to select the explanatory variables^41,65^. We have carried out the estimations on both the full and post-LASSO extended sets, with both specifications having similar performance. For simplicity, we only display the results for the approach that performed better in each individual analysis.

### Assessing model performance

We evaluate model performance using data (the ‘test set’) that was unseen by the model during the training stage. In this way, we can evaluate each model’s out-of-sample prediction quality. We first compare the performance of the OLS model to those of two ML algorithms, using the restricted set of variables that are standard in the wellbeing literature; we then carry out an analogous comparison for the extended set of individual characteristics.

As we evaluate model performance by out-of-sample prediction, any performance improvements from the more-flexible ML framework or the extended set of variables is not a mechanical result of overfitting. The improvements we observe, therefore, genuinely indicate that the literature’s standard regression models do not utilise all of the relevant information contained in social surveys.

We should not of course expect all of the observed variance in reported wellbeing to be predictable. For example, responses to wellbeing questions can be influenced by random and extraneous factors that are not relevant for global evaluative wellbeing, such as passing moods or social desirability. With this in mind, we should interpret reported wellbeing levels as a combination of a potentially-predictable latent state and a measurement error (see Refs.^66,67^ for a similar approach). Our aim should therefore be to successfully predict the share of variance in reported wellbeing that can be attributed to a respondent’s latent state. Following^46^, we approximate this share of the explainable variance by the test-retest correlation in reported wellbeing (see^68^ for a formal derivation). In particular, Ref.^46^ find a test-retest correlation of 0.59 for life satisfaction, which is in line with earlier findings on smaller samples (see e.g.^69^). We take this as an upper bound for any model’s ability to predict wellbeing.

### Assessing variable importance

To answer our second research question, we need to establish the importance of each explanatory variable in predicting wellbeing. We do so in two ways.

We first use *permutation importances* (PIs) to measure the degree to which each algorithm relies on a given variable in making its predictions^70^. PIs are calculated by randomly shuffling a given variable’s observed values across individuals in the test data and evaluating the extent to which the predictive performance (in terms of R-squared) of a given algorithm falls when the variable’s values are permuted in this way. This operation is carried out 10 times. The reported PI is the average change in the R-squared across these 10 iterations. The greater the average drop in the R-squared, the more important is the variable in predicting wellbeing.

To understand the direction of the variables’ effects we also report *pseudo partial effects* (PPEs). These are calculated by taking the difference in predicted wellbeing after setting each explanatory variable to a given set of values. Specifically, for continuous and ordinal variables we set the variable to the third and first quartile of their distributions and then calculate the mean difference in predicted wellbeing. For binary variables (including the dummies calculated from the categorical variables), we predict wellbeing when setting each individual’s value to either 0 or 1.

A key advantage of PIs and PPEs is that they can be used with any kind of algorithm, allowing us to compare the way in which each algorithm makes use of the available data.

## Supplementary Information

Supplementary Information.

## Data Availability

This work is based on secondary data sourced from the German Socio-Economic Panel (SOEP), the United Kingdom Household Longitudinal Household Survey (UKHLS), and the Gallup Daily Poll (Gallup). The SOEP is accessible for academic researchers upon signing a data distribution contract. Legal access can be obtained via the DIW Berlin (see: https://www.diw.de/en/diw_01.c.601584.en/data_access.html). The UKHLS can be accessed via the UK Data Archive (see: https://www.understandingsociety.ac.uk/documentation/access-data) following registration. The Gallup data is not publicly accessible. However, it may be possible to obtain the data from Gallup Analytics for replication purposes. We will of course share our specific data-files for any authors who have obtained legal access to these datasets. Please contact Caspar Kaiser (caspar.kaiser@wbs.ac.uk) for any such requests.
