# Supplementary material for: Machine learning in the prediction of human wellbeing
Source: Sci Rep. 2025 Jan 10;15:1632. doi: 10.1038/s41598-024-84137-1 (PMC11723942; doi:10.1038/s41598-024-84137-1)
Supplement: Supplementary file 1 — Supplementary Information. [file 41598_2024_84137_MOESM1_ESM.pdf]

Online Appendix for: Machine Learning in the Prediction of Human Wellbeing

Ekaterina Oparina ⓡ (London School of Economics)
Caspar Kaiser ⓡ (Warwick Business School & University of Oxford)
Niccolò Gentile ⓡ (University of Luxembourg)
Alexandre Tkatchenko (University of Luxembourg)
Andrew E. Clark (PSE - CNRS)
Jan-Emmanuel De Neve (University of Oxford)
Conchita D'Ambrosio (University of Luxembourg)

November 20, 2024

A Additional discussion

A.1 Results for positive and negative affect

We have also evaluated the performance of gradient boosting and random forests on measures of positive and negative affect. The findings for evaluative wellbeing discussed in the main text generalise to these measures. In the 2013 Gallup data, positive affect is measured by the average figure from dummy variables indicating whether the respondent felt happiness or joy, or smiled during the previous day. Negative affect is calculated analogously from dummies indicating pain, worry, sadness and anger. In the German SOEP, positive affect is the self-reported frequency of being happy over the past 4 weeks (on a 1 to 5 scale), and negative affect as the analogous average of being angry, sad or worried. The UKHLS dataset does not contain comparable affect data and is not used in this part of analysis.

The detailed Gallup results appear in the top panels of Figure A4 and Table A7. It is striking that negative affect is easier to predict than positive affect. This finding holds across algorithms, with R-squared figures ranging from 0.423 to 0.464 for negative affect, and between 0.261 and 0.296 for positive affect. Random forests and gradient boosting outperform both OLS and LASSO. As was the case for life evaluations, gradient boosting performs the best, with gains in R-squared over OLS of 0.041 for negative affect and 0.036 for positive affect. Regarding variable importance, Table A7 shows that good health is even more important for predicting positive and negative affect in the Gallup data than it was for life evaluation. Moreover, in line with previous work (e.g. Kahneman and Deaton [1]), variables relating to material conditions – like income – do not feature in the set of the most-important variables when modelling affect.

Our results are qualitatively similar in the German data: gradient boosting performs best, and positive affect is harder to predict than negative affect (see Online Appendix Table A8 and the bottom panels of Figure A4.

A.2 Results for panel data

Our main findings regarding the ML estimation of levels in evaluative wellbeing are robust to exploiting the panel dimension of the German SOEP and the UKHLS. As there is no standard procedure for the introduction of individual fixed effects in the ML algorithms that we use, we implement an approach similar to the Mundlak correction for linear models [2, 3]: we pool all years of the UKHLS and SOEP data, demean all covariates at the individual level and include both an individual's average value over time of each covariate as well as their year-specific deviations from their individual mean. The demeaned level of wellbeing is the dependent variable.

The relative predictive performance of OLS and ML in the panel specification is similar to that in the cross-section analysis for the individual years. In the UKHLS, the OLS R-squared is 0.140. The use of RF produces a small improvement, with the R-squared increasing to 0.143. Gradient boosting provides a further improvement, yielding an R-squared of 0.150. In

the German SOEP, the OLS R-Squared is 0.122, with once again both the random forest and gradient boosting leading to better R-Squared figures of, respectively, 0.150 and 0.156. As shown in Tables A9 and A10, the most-important variables predicting wellbeing are almost exclusively the average values of the individual covariates. One exception in both the UKHLS and SOEP is the *Health limits activities* variable. As such, deviations in individual health status (from their average value) seem to be important in predicting wellbeing.

Finally, in further analyses not shown we also predicted individual-level changes in wellbeing from one wave to another. Again, random forests and (especially) gradient boosting outperform OLS by a moderate amount.

A.3 Further discussion of estimation methods

The OLS estimates are the solution to the problem $\arg\min_b \sum_{i=1}^{N}(x_i'b - s_i)^2$. Here, x_i is a vector of explanatory variables and b the vector of coefficients. The wellbeing of respondent i is denoted by s_i. In turn, LASSO estimates are the solution to $\arg\min_b \sum_{i=1}^{N}(x_i'b - s_i)^2 + \lambda \sum_{k=1}^{K} |b_k|$. Here, λ is a hyperparameter, the preferred value of which is found using a grid search. LASSO and OLS are equivalent for $\lambda = 0$.

As noted in the main text, Random Forests and Gradient Boosting are algorithms based on regression trees. Regression trees are generated via a recursive binary splitting algorithm. The algorithm splits the sample along values of covariates and predicts the outcome in each subsample, or *node*, as the mean outcome within each node. More formally, at each step k, the data D is split into two nodes $D_{L,k}$ and $D_{R,k}$. The location of the split within the data is determined by some variable x_j and an associated threshold $\tau_{k,j}$. The nodes $D_{L,k}$ and $D_{R,k}$ are defined as in [4]: $D_{(L,k)} = \{x|x_j < \tau_{k,j}\}; D_{(R,k)} = \{x|x_j \geq \tau_{k,j}.\}$. The predicted values are the mean value of s within each node, *i.e.* $\hat{s}_{D_{m,k}} = N_{D_{m,k}}^{-1} \sum_{i:X_i \in D_{m,k}} s_i$, for $m \in \{L, R\}$, where $N_{D_{m,k}}$ is the number of respondents in each node. At each step, the splitting variable x_j and the threshold $\tau_{k,j}$ are determined by minimising the residual sum of squares at either side of the split. The nodes $D_{L,k}$ and $D_{R,k}$ are in turn used as inputs for the next step. This procedure is repeated until some final number of *leaves* is found. By construction, every split reduces the mean squared error (MSE). If the size of the tree is not limited, the algorithm will overfit the data in the training set. This issue can be tackled by aggregating the predictions from multiple smaller trees. Random forests and gradient boosting are both examples of this strategy [4].

When using Gradient Boosting, we begin with the predictions $\hat{s}_{T_1}$ of a first tree T_1 and calculate the residual $\hat{s}_{T_1} - s_i = e_{T_1}$. A second tree T_2 is then fitted on these residuals to obtain predicted residuals, $\hat{e}_{T_1}$. The overall predictions are then given by $\hat{s}_{T_1} + \hat{e}_{T_1} = \hat{s}_{T_2}$. This process is repeated *Ntrees* times, producing increasingly accurate predictions of s. Since gradient-boosted collections of trees overfit in the training set with large *Ntrees*, we select this hyperparameter via a grid search. To further reduce overfitting, the size of the update at each step is reduced by adding a penalty $0 < \gamma \leq 1$, and predictions are updated with the rule $\hat{s}_{T_k} + \gamma \hat{e}_{Tk} = \hat{s}_{Tk+1}$. The penalty γ is also selected via a grid search. We here use a standard

implementation of gradient boosting. We also evaluated the performance of extreme gradient boosting (XGBoost [5]), which yielded only negligible improvements.

Appendix Tables and Figures

Table A1: The optimal hyperparameters used in the extended specifications (post-LASSO extended specification in parentheses)

	**Panel A: Random Forest**		
	SOEP	Gallup	UKHLS
MaxDepth	96 (70)	70 (70)	30 (20)
Nvars	225 (65)	80 (80)	400 (130)
Ntrees	1000 (1000)	1000 (1000)	1000 (1000)
MinLeaf	1 (1)	5 (5)	15 (5)
	**Panel B: Gradient Boosting**		
	SOEP	Gallup	UKHLS
MaxDepth	8 (8)	3 (3)	5 (7)
Nvars	75 (30)	40 (40)	100 (30)
Ntrees	6000 (2000)	16000 (16000)	2000 (2000)
MinLeaf	1 (1)	1 (1)	1 (1)
Learning rate (γ)	0.005 (0.01)	0.0063 (0.0063)	0.01 (0.01)

Notes: The hyperparameters are identified via a grid search by minimising the average MSE across 4 folds of cross-validation. *MaxDepth* is the maximum depth of each branch of each tree. *Nvars* is the maximum number of randomly-picked variables used to perform splits within each tree. *MinLeaf* is the minimum number of training individuals that must be in each leaf of a given tree (fixed to 1 for gradient boosting). *Ntrees* is the number of trees fitted (fixed to 1,000 for random forests). The learning rate (γ) is the rate at which predictions are updated (only applicable to gradient boosting).

Table A2: List of variables in the restricted set: *min - max, mean (sd).*

Variable	SOEP	UKHLS	Gallup
Age	16 - 105	18 - 103	18 - 99
	47.08 (17.27)	49.32 (17.74)	52.74 (18.08)
Area of residence	16 distinct values	12 regions	51 distinct values
BMI	11.10 - 84.50	11.80 - 74.20	10.62 - 114.17
	26.33 (4.53)	26.33 (3.19)	27.40 (5.66)
Disability status	Binary	Binary	n.a.
Education	18 - 7 (years of education)	6 distinct values	6 distinct values
Labour-force status	Binary	12 distinct values	4 distinct values
Log HH income	0 - 13.88	-0.80 - 12.52	3.40 - 9.90
equiv. in UKHLS and SOEP	9.94 (0.67)	7.40 (0.70)	8.39 (1.02)
Ethnicity/Migration background	3 distinct values (migration background)	18 distinct values (ethnicity)	5 distinct values (ethnicity)
Health	0 – 396 (doctor visits in prev. year) 0.12 (0.33)	Health limits activities (3 distinct values)	Binary (self-assessed health problems)
Housing status	4 distinct values	6 distinct values	n.a.
Marital status	5 distinct values	10 distinct values	6 distinct values
Month of interview	12 distinct values	24 distinct values	12 distinct values
Number of children in HH	0 - 11	0 - 9	0 - 15
	0.85 (1.19)	0.56 (0.95)	0.55 (1.05)
Number of people in HH	1 - 16	1 - 16	n.a.
	2.99 (1.52)	2.83 (1.46)	n.a.
Religion	10 distinct values	Binary	8 distinct values
Sex	Binary	Binary	Binary
Working hours	0 - 6669	0 - 180	4 distinct values
	1033.73 (1074.71)	21.13 (20.81)	

Notes: For continuous variables, the range is reported. For SOEP, the values for the categorical variables are as follows. *Area of residence*: Each of the 16 Bundesländer. *Ethnicity/Migration background:* No migration background, Direct migration background, Indirect migration background. *Housing status:* Main Tenant, Sub-Tenant, Owner, Nursing Home/ Retirement Community. *Marital status:* Married, Single,

Widowed, Separated, Divorced. *Religion:* Catholic, Protestant, Christian Orthodox, Other Christian, Muslim, Muslim (Shiite), Muslim (Sunnite), Muslim (Alevite), Other, No religion. For UKHLS, the values for the categorical variables are as follows. *Area of residence:* North East, North West, Yorkshire and Humberside, East Midlands, West Midlands, East of England, London, South East, South West, Wales, Scotland, Northern Ireland. *Education:* Degree, Other higher degree, A-level etc, GCSE etc., Other qualification, No qualifications. *Labour-force status:* Self-employed, Paid employment (FT/LT), Unemployed, Retired, On maternity leave, Family care or home, Full-time student, LT sick or disabled, Govt training scheme, Unpaid, family business, On apprenticeship, Doing something else. *Ethnicity:* British/English/Scottish/Welsh/Northern Irish, Irish, Gypsy or Irish traveller, Any other White background, White and Black Caribbean, White and Black African, White and Asian, Any other mixed background, Indian, Pakistani, Bangladeshi, Chinese, Any other Asian background, Caribbean, African, Any other Black background, Arab, Any other ethnic group. *Health limits moderate activities:* Yes, a lot; Yes, a little; No, not at all. *Housing status:* Owned outright, Owned/being bought on mortgage, Shared ownership (part-owned part-rented), Rented, Rent free, Other. *Marital status:* Single and never married/in civil partnership, Married, In a registered same-sex civil partnership, Separated but legally married, Divorced, Widowed, Separated from civil partner, A former civil partner, A surviving civil partner, Living as couple. For Gallup, the values for the categorical variables are as follows. *Area of residence:* 51 States. *Education:* Less than high school, High school, Technical/Vocational school, Some college, College graduate, Post-graduate. *Labour-force status:* Employed, Self-employed, Employed and self-employed, not employed. *Ethnicity:* White, Other, Black, Asian, Hispanic. *Marital status:* Single, Married, Separated, Divorced, Widowed, Living with partner (not married). *Religion:* Protestant, Catholic, Jewish, Muslim, Mormon, Other Christian, Other, No religion. *Working hours:* 30 or more hours per week, 15 to 29 hours per week, 5 to 14 hours per week, less than 5 hours per week.

Table A3: List of variables in the extended set.

Group	Description		
	SOEP	UKHLS	Gallup
Area	State of residence.	Country of residence, government office region, urban or rural area.	State of residence.
Cognitive skills		Numeric ability, verbal fluency and word recall scores; self-rated memory, interviewer rated language ability and anxiety.	
Education	Education level, currently in education, need training.	Educational qualifications, age of leaving school and further education, additional training.	Learn something every day, education.
Employment	Employment status, hours worked, current job characteristics.	Current and past employment characteristics, including full- or part-time employment, number of jobs, hours worked, industry, socio-economic classification, unemployment spells, location and commute.	Employment status, working hours, work environment, occupation, company and supervisor characteristics.
Finances	Income from different sources, entitlement to other forms of allowances, dept and assets.	Incomes from various sources, spendings on energy, food and alcohol, problems paying for housing, bills or council tax.	Income, basic access index, not enough money for food or shelter.
Friends and socialising	Number of close friends, use of social website, going out socially.	Number of close friends and their characteristics, belonging to social website, going out socially.	Treated with respect.
Health	Physical and mental health conditions, hospital stays, eventual limitations, and behaviours, including sleep, smoking and diet.	BMI, health conditions, health limits activities.	BMI, health conditions, doctor visits, pain, health limits activities, health insurance and health behaviours, including smoking and diet.
Household composition and family relationships	Household composition, marital status, relationship with other generations in the family, leisure and housework.	Household composition, marital status, family members outside of the household, providing or receiving help, caring responsibilities, quality of the relationships with the partner.	Number of children, marital status.
Interview	Month of interview.	Interview characteristics, including month, year, language of the interview, respondent's cooperation and understating, other people present.	Month, day of the week, time zone.
Membership in organisations		Being a member and being active in organisations, including political party, professional organisations, community groups, social or sports clubs, trade unions or others.	Member of labour union, served in the U.S. military.
Neighbourhood	Noise and pollution, quality of area, relationships with neighbours, local amenities.	Tenure in the neighbourhood and the neighbourhood characteristics, including safety, standards of local services, social cohesion, trust, interactions with neighbours.	City and area characteristics, including safety, affordability of food, medicine and places for exercise, appreciation of the city/area.
News sources		Reported news sources, including TV, internet, newspapers and others; most frequent TV channel, hours of TV per week.	
Personality traits	BIG 5 personality traits, risk preferences.	BIG 5 personality traits.	
Residence	Home ownership and characteristics of the dwelling, including the items in the accommodation.	Characteristics of the residence, including the items in the accommodation (e.g. television, washing machine, etc.), number of bedrooms, value of property; past and current tenancy status.	
Socio-demographic characteristics	Age, ethnicity, gender, nationality, religion.	Age, sex, ethnic group, migration status, religion, parents' education, ethnic group and county of origin.	Age, race, gender, religion.
Views and beliefs	Political preferences, importance of being able to afford something, having children, helping others, being socially and politically active.	Sense of civic duty, attitudes towards voting, perceived political influence, level of interest in politics, partisan support.	State of current economy, national economy is getting better.

Notes: The full list of variables is available here: https://data.mendeley.com/datasets/pgrvssrwy6.

Table A4: Permutation Importance (PI) and Pseudo Partial Effects (PPE) in OLS, RF and GB on the Extended Set of variables: the 10 most-important variables.

	OLS			Random forest			Gradient boosting		
	Variable name	PI	PPE	Variable name	PI	PPE	Variable name	PI	PPE
				**Panel A: SOEP**					
1	Health limits daily life: a lot	.029	-.780	Health limits social life	.032	.154	Health limits social life	.022	.172
2	Worry a lot	.025	-.146	Health limits daily life: a lot	.028	-.742	Worry a lot	.021	-.100
3	Health limits social life	.023	.187	Worry a lot	.020	-.113	Health limits daily life: a lot	.019	-.628
4	Personal patience	.011	.129	Equiv. HH income	.018	.202	Personal patience	.010	.174
5	Health limits daily life: a bit	.009	-.266	Deal well with stress	.015	.160	Deal well with stress	.008	.128
6	Partner in HH	.008	.222	Personal patience	.008	.106	Health limits daily life: a bit	.006	-.220
7	No monthly savings	.008	-.186	No annual holiday trip	.007	-.114	Partner in HH	.006	.152
8	Deal well with stress	.006	.080	No monthly savings	.007	-.110	Risk tolerance	.006	.036
9	House needs repair	.005	-.126	Not unemployed	.006	.303	Equiv. HH income	.006	.152
10	Hours of sleep on workday	.004	.077	Unemployment benefit	.005	-000	Number of doctor visits	.006	-.086
				**Panel B: UKHLS**					
1	Regret getting married	.032	.418	Worries a lot (Big 5)	.030	-.146	Worries a lot (Big 5)	.033	-.188
2	Worries a lot (Big 5)	.029	-.274	Feeling relaxed (Big 5)	.027	.238	Feeling relaxed (Big 5)	.019	.212
3	Feeling relaxed (Big 5)	.016	.240	Health limits kind of work	.009	.040	Regret getting married	.011	.209
4	Kiss partner	.012	-.218	Belong to neighbourhood	.009	-.179	Does a thorough job (Big5)	.008	.069
5	Does thorough job (Big 5)	.006	.112	Age squared	.009	.007	Kiss partner	.007	-.110
6	Share interests w/ partner	.006	-.161	Regret getting married	.009	.137	Age squared	.007	.002
7	Belong to neighbourhood	.005	-.107	Health limits work amount	.008	.032	Health limits kind of work	.007	.053
8	Sociable (Big 5)	.005	.094	Does thorough job (Big 5)	.007	.053	Health limits work amount	.006	.049
9	Health limits work amount	.005	.070	Consider divorce (never)	.006	.106	Belong to neighbourhood	.006	-.162
10	Long term sick or disabled	.005	-.420	Sociable (Big 5)	.006	.081	Sociable (Big 5)	.006	.126
				**Panel C: Gallup**					
1	Learn something every day	.031	.43	Learn something every day	.033	.34	Learn something every day	.028	.35
2	City/area is perfect	.021	.32	City/area is perfect	.026	.42	City/area is perfect	.021	.39
3	HH income	.013	.15	HH income	.021	.30	HH income	.018	.26
4	Economy in this country	.013	.21	Cannot afford healthcare	.021	-.54	Health index	.015	.16
5	Cannot afford healthcare	.010	-.38	Economy in this country	.015	.21	Economy in this country	.015	.22
6	Health limits activities	.010	-.04	Physical health index	.013	.15	Cannot afford healthcare	.013	-.40
7	Health encouragement	.010	.12	Health limits activities	.010	-.03	Health encouragement	.008	.17
8	Physical health index	.010	.14	Health encouragement	.010	.17	Health limits activities	.008	-.01
9	Female	.008	.24	Female	.005	.13	Age and age-squared	.005	.03
10	Ever diag. w/ depression	.008	-.28	Ever diag. w/ depression	.005	-.16	Female	.005	.25

Notes: The following variables are shown. SOEP: Dummies: *Health limits daily life a lot*, *Health limits daily life a bit*, *Partner in HH*, *No monthly savings*, *Not unemployed*, *No emergency reserves*, and *No annual holiday trip.* Likert scales: *Limited socially due to health* (1 – always to 5 – never), *Worries a lot* and *Deals well with stress* (1 – not at all to 7 – totally agree), *Personal patience* (0 – very bad to 10 – very good), *House needs repair* (1 – in good condition, 3 – needs major renovation). Continuous: *Equiv. HH income*, *Hours of sleep*, *Number of Doctor visits*, *Risk Tolerance and Unemployment Benefit.* UKHLS: Dummies: *Health not limiting activities.* Likert scales: *Pain interferes with work* (1 – not at all to 5 – extremely), *Regret getting married*, *Share interests with partner*, *Consider divorce* and *Kiss partner* (1 – all the time, 6 – never), *Health limits work amount* and *Health limits kind of work* (1 – all of the time, 5 – none of the time); Big 5 traits, including *Worries a lot*, *Feeling relaxed*, *Does thorough job*, *Is sociable* (1 – does not apply to 7 – applies perfectly), *Belong to neighbourhood* (1 – strongly agree – 5 strongly disagree). Continuous: *Age squared.* Gallup: Dummies: *Cannot afford healthcare*, *Female*, *Ever diagnosed with depression.* Likert scales: *Learn something every day*, *City/area is perfect* and *Receives Health encouragement* (1 – strongly disagree, 5 – strongly agree), *Economy in this country* (1 – poor to 4 – Excellent), *Health limits activities in the last month* (0 to 30 days). Continuous: *Age*, *age squared*, *HH income*, *Physical health index.*

Table A5: Correlations between the Permutation Importance ranks in different algorithms.

	OLS *vs.* GB	OLS *vs.* RF	GB *vs.* RF
SOEP	0.70 (p<0.000; t=9.77)	0.58 (p<0.000; t=7.03)	0.79 (p<0.000; t=12.60)
UKHLS	0.74 (p<0.000; t=11.03)	0.68 (p<0.000; t=9.12)	0.86 (p<0.000; t=16.77)
Gallup	0.85 (p<0.000; t=16.31)	0.70 (p<0.000; t=9.57)	0.81 (p<0.000; t=13.90)

Notes: The correlation figures refer to the Top-100 variables (using the OLS ranking). These are Spearman rank correlations. N=100 and df=98 throughout. P-values are taken from two-sided hypothesis tests.

Table A6: Permutation Importance (PI) and Pseudo Partial Effect (PPE) in OLS, RF and GB on the Restricted Set of variables: the 10 most-important variables.

	OLS			Random forest			Gradient boosting		
	Variable name	PI	PPE	Variable name	PI	PPE	Variable name	PI	PPE
				**Panel A: SOEP**					
1	Age and age-squared	.10	-1.70	Equiv. HH Income	.13	.27	Equiv. HH Income	.14	.46
2	Equiv. HH Income	.10	.26	Age and age-squared	.12	-.14	Age and age-squared	.13	-.18
3	Number of doctor visits	.08	-.14	Number of doctor visits	.11	-.28	Number of doctor visits	.12	-.63
4	Marital Status - Single	.07	-.40	Disability Status	.04	-.40	Disability Status	.03	-.45
5	No. of children in HH	.06	.30	No. of children in HH	.03	.07	Working hours	.02	-.29
6	Disability Status	.04	-.52	No. of people in HH	.03	.02	No. of years of education	.02	.17
7	No. of people in HH	.03	-.17	No. of years of education	.02	.07	No. of children in the HH	.02	.08
8	No. of years of education	.03	.11	House Ownership: Owner	.02	.12	No. of people in HH	.02	-.16
9	Marital Status – Divorced	.02	-.38	Working hours	.01	.04	Marital Status – Single	.02	-.19
10	Marital Status - Separated	.02	-.74	BMI	.01	-.02	Marital Status - Separated	.01	-.53
				**Panel B: UKHLS**					
1	Health limits activities: a lot	.024	-.670	Age	.040	.052	LT sick or disabled (empl.)	.018	-.587
2	Single	.020	-.336	Equiv. HH income	.015	.161	Age	.015	.052
3	LT sick or disabled (empl.)	.017	-.797	Health limits activities: a lot	.014	-.377	Health limits activities: a lot	.012	-.377
4	Age	.018	.015	Not disabled (health)	.014	.215	Not disabled (health)	.010	.215
5	Health limits activities: a bit	.014	-.327	Health limits activities: a bit	.012	-.226	Renting house	.007	-.106
6	Not disabled (health)	.011	.240	LT sick or disabled (empl.)	.011	-.587	Health limits activities: a bit	.007	-.226
7	Retired	.010	.235	Unemployed	.006	-.193	Equiv. HH income	.006	.161
8	Renting house	.008	-.208	Renting house	.005	-.106	Unemployed	.006	-.193
9	Unemployed	.008	-.343	Single	.005	-.136	Retired	.005	.099
10	Equiv. HH income	.008	.083	Retired	.003	.099	Single	.003	-.136
				**Panel C: Gallup**					
1	Health limits activities	.064	.84	HH income	.062	.48	HH income	.067	.48
2	HH income	.049	.30	Health limits activities	.057	.69	Health limits activities	.054	.71
3	Post-graduate education	.026	.58	Age and age-squared	.046	.43	Age and age-squared	.041	.44
4	Married	.013	.33	Married	.013	.26	Married	.013	.27
5	College Graduate	.010	.37	Female	.010	.23	Female	.013	.29
6	Female	.010	.29	Post-graduate education	.008	.43	Post-graduate education	.008	.34
7	Age and age-squared	.008	.24	BMI	.005	.29	BMI	.005	-.12
8	Hispanic	.003	.28	Working Hours Missing	.005	-.12	Hispanic	.003	.15
9	Atheist	.003	-.19	Hispanic	.003	.06	Black	.003	.10
10	High school graduate	.003	.17	Asian	.003	.02	Working Hours Missing	.003	-.06

Note: The total set of variables available in the restricted set appears in Table A2.

Table A7: Permutation Importance (PI) and Pseudo Partial Effect (PPE) in OLS, RF and GB for positive and negative affect: the 10 most-important variables (using 2013 Gallup data with the Extended Set of variables).

	OLS			Random forest			Gradient boosting		
	Variable name	PI	PPE	Variable name	PI	PPE	Variable name	PI	PPE
				**Panel A: Positive affect**					
1	Age	.14	-.26	Physical health index	.07	.42	Physical health index	.16	.62
2	Age squared	.09	-.26	Learn something every day	.06	.43	Learn something every day	.05	.49
3	Physical health index	.09	.66	Not treated with respect	.03	-1.39	Not treated with respect	.03	-1.13
4	Learn something every day	.05	.82	Health encouragement	.02	.13	Health encouragement	.02	.14
5	Not treated with respect	.03	-1.52	Diagnosed w. depression	.01	.27	BMI	.01	.02
6	Health encouragement	.02	.23	City/area is perfect	.00	.17	Diagnosed w/ depression	.01	.34
7	In workforce	.01	.44	Health limits activities	.00	-.01	Has any health problems	.01	-.26
8	Diagnosed w/ depression	.01	.52	BMI	.00	.09	City/area is perfect	.00	.17
9	Not working	.00	-.32	Age squared	.00	-.11	Health limits activities	.00	.21
10	Tuesday	.00	-.33	Age	.00	-.11	Female	.00	.20
				**Panel B: Negative affect**					
1	Physical health index	.26	-.11	Physical health index	.31	-.15	Physical health index	.50	-.18
2	Not treated with respect	.03	.16	Not treated with respect	.04	.17	BMI	.04	-.02
3	Diagnosed w/ depression	.02	-.09	BMI	.03	-.01	Not treated with respect	.03	.15
4	Age squared	.01	-.03	Diagnosed w. depression	.02	-.07	Has any health problems	.02	.06
5	BMI	.01	-.03	Health limits activities	.01	-.02	Diagnosed w/ depression	.02	-.07
6	Has any health problems	.01	.04	Has any health problems	.01	.02	Health limits activities	.02	-.06
7	Cannot afford healthcare	.01	-.05	Cannot afford healthcare	.01	-.04	Had a cold yesterday	.01	.07
8	Wednesday	.00	.05	City/area is perfect	.00	-.02	Cannot afford healthcare	.01	-.04
9	Neck or backpain	.00	-.03	Neck or backpain	.00	-.02	Headache yesterday	.00	.02
10	Time Zone E	.00	.03	Age	.00	-.04	City/area is perfect	.00	-.02

Notes: The following variables are shown. Dummies: *Cannot afford healthcare*, *Female*, *Ever diagnosed with depression*, *Not treated with respect*, *In workforce*, *Has any health problems*, *Tuesday*, *Wednesday*, *Neck or backpain*, *Time Zone E.* Likert scales: *Learn something every day*, *City/area is perfect*, *Receives Health encouragement* (1 – strongly disagree, 5 – strongly agree), *Economy in this country* (1 – poor to 4 – Excellent), *Health limits activities in the last month* (0 to 30 days). Continuous: *Age*, *age squared*, *Log HH income*, *Physical health index.*

Table A8: Permutation Importance (PI) and Pseudo Partial Effect (PPE) of OLS, RF and GB for positive and negative affect of the 10 most-important variables (using 2013 SOEP data with the Extended Set of variables).

	OLS			Random forest			Gradient boosting		
	Variable name	PI	PPE	Variable name	PI	PPE	Variable name	PI	PPE
				**Panel A: Positive affect**					
1	Partner in HH	.03	.21	Partner in HH	.03	.17	Partner in HH	.03	.17
2	Worry a lot	.02	-.05	Health limits social life	.02	.03	Worry a lot	.02	-.03
3	Health limits social life	.02	.08	Number of close friends	.01	.07	Health limits social life	.01	.05
4	Deal well with stress	.01	.04	Worry a lot	.01	-.02	Number of close friends	.01	.08
5	Excursions/short trips	.01	-.07	Deal well with stress	.01	.04	Deal well with stress	.01	.04
6	Number of close friends	.01	.04	Excursions/short trips	.01	-.03	Excursions/short trips	.01	-.06
7	Last Word Fin. Decisions-NA	.01	-.08	HH income	.00	.04	Hours of childcare per day	.00	.01
8	Importance: to help others	.01	-.07	Attend cinema/concerts	.00	-.04	Use of social networks	.00	-.06
9	Health limits daily life: a lot	.00	-.12	Am sociable	.00	.01	Importance to help others	.00	-.05
10	Psychiatric problems	.00	-.16	Visit neighbours/friends	.00	-.01	Personal patience	.00	.04
				**Panel B: Negative affect**					
1	Worry a lot	.11	.13	Worry a lot	.13	.03	Worry a lot	.12	.05
2	Health limits social life	.04	-.12	Health limits social life	.04	-.08	Health limits social life	.04	-.12
3	Female	.03	.20	Deal well with stress	.03	-.03	Female	.02	.16
4	Deal well with stress	.02	-.06	Female	.02	.15	Deal well with stress	.02	-.03
5	Hours of sleep	.01	-.10	Psychiatric problems	.01	.18	Number of doctor visits	.01	.08
6	Health limits daily life: a lot	.01	.18	Number of doctor visits	.01	.05	Hours of sleep	.01	-.07
7	Psychiatric problems	.01	.25	Hours of sleep	.01	-.04	Psychiatric problems	.01	.22
8	Personal patience	.01	-.06	Annual pension	.01	.00	Personal Patience	.01	-.05
9	Health affects tiring tasks	.01	.15	Personal Patience	.01	-.03	Annual pension	.01	.00
10	Number of doctor visits	.01	.03	Physical pain last 4 weeks	.00	-.03	Health limits daily life: a lot	.00	.12

Notes: The following variables are shown.: Dummies: *Health limits daily life a lot*, *Health limits daily life a bit*, *Partner in HH*, *No monthly savings*, *Not unemployed*, *No emergency reserves*, *Last word in financial decisions-NA*, *Psychiatric problems*, *Female*, and *No annual holiday trip*. Likert scales: *Limited socially due to health* (1 – always to 5 – never), *Worries a lot*, *Importance: To help others* (1 – Very Important to 4 – Not important), *Deals well with stress* (1 – not at all to 7 – totally agree), *Personal patience* (0 – very bad to 10 – very good), *House needs repair* (1 – in good condition, 3 – needs major renovation), *Attend cinema/concerts* (1 – Daily to 4 - Infrequent), *Am Sociable* (1 to 7), *Visit neighbours/friends* (1 – Daily to 5 - Never), *Use of social networks* (1 – Daily to 5 - Never), *Health affects tiring tasks* (1 – A lot to 3 - Not at all), and *Physical pain last 4 weeks* (1 – Always to 5 - Never). Continuous: *Log HH income*, *Hours of sleep*, *Number of doctor visits*, *Risk tolerance*, *Unemployment benefit*, *Excursions/short trips*, *Number of close friends*, *Hours of childcare per day*, *Annual pension.*

Table A9: Permutation Importance (PI) of OLS, RF and GB for levels of wellbeing of the 10 most-important variables (using pooled UKHLS data with the Restricted Set of variables). For each covariate, the models include the average value and the annual deviation from that average.

	OLS		Random forest		Gradient boosting	
	Variable name	PI	Variable name	PI	Variable name	PI
1	Health limits activities: a lot (avg.)	.041	Age (avg.)	.025	Age (avg.)	.026
2	Not disabled (health) (avg.)	.020	Not disabled (health) (avg.)	.020	Not disabled (health) (avg.)	.022
3	Married (avg.)	.019	Health limits activities: a lot (avg.)	.018	Health limits activities: a lot (avg.)	.021
4	Health limits activities: a bit (avg.)	.017	Health limits activities: a bit (avg.)	.014	Health limits activities: a bit (avg.)	.014
5	LT sick or disabled (empl.) (avg.)	.015	LT sick or disabled (empl.) (avg.)	.011	Equiv. HH income (avg.)	.012
6	Age (avg.)	.013	Equiv. HH income (avg.)	.009	LT sick or disabled (empl.) (avg.)	.012
7	Retired (avg.)	.012	Married (avg.)	.006	Married (avg.)	.009
8	Equiv. HH income (avg.)	.010	Retired (avg.)	.005	Retired (avg.)	.006
9	Unemployed (avg.)	.007	Unemployed (avg.)	.004	Unemployed (avg.)	.005
10	Rents the house/flat	.005	Health limits activities: a bit	.003	Health limits activities: a lot	.004

Notes: All covariates apart from month, ethnicity and sex are split into individual means and deviation from the mean. Individual averages are denoted by *(avg.)*; variables without additional notes are the deviations from the individual means.

Table A10: Permutation Importance (PI) of OLS, RF and GB for deviations from the average wellbeing and individual level of wellbeing of the 10 most-important variables (using pooled SOEP data with the Restricted Set of variables). For each covariate, the models include the average value and the annual deviation from that average.

	OLS		Random forest		Gradient boosting	
	Variable name	PI	Variable name	PI	Variable name	PI
1	Age (avg.)	.082	Age (avg.)	.126	Age (avg.)	.124
2	Number of doctor visits (avg.)	.039	Equiv. HH Income (avg.)	.059	Equiv. HH Income (avg.)	.049
3	Equiv. HH Income (avg.)	.039	Number of doctor visits (avg.)	.041	Number of doctor visits (avg.)	.042
4	No. of children in the hh (avg.)	.025	Not disabled (health) (avg.)	.021	Not disabled (health) (avg.)	.016
5	Not disabled (health) (avg.)	.016	No. of people in hh (avg)	.014	Age	.010
6	Single (avg.)	.016	No. of children in hh (avg.)	.011	No. of people in hh (avg.)	.009
7	Divorced (avg.)	.007	House Owner	.009	No. of children in hh (avg.)	.008
8	No. of people in hh (avg.)	.006	Age	.008	Number of doctor visits	.007
9	Number of doctor visits	.005	Number of doctor visits	.005	Single	.006
10	House Owner	.005	Number of years of education	.005	House Owner	.006

Notes: All covariates apart from month, ethnicity and sex are split into individual means and deviation from the mean. Individual averages are denoted by *(avg.)*; variables without additional notes are the deviations from the individual means.

Figure A1: Histograms of life satisfaction for SOEP, UKHLS and Gallup data.

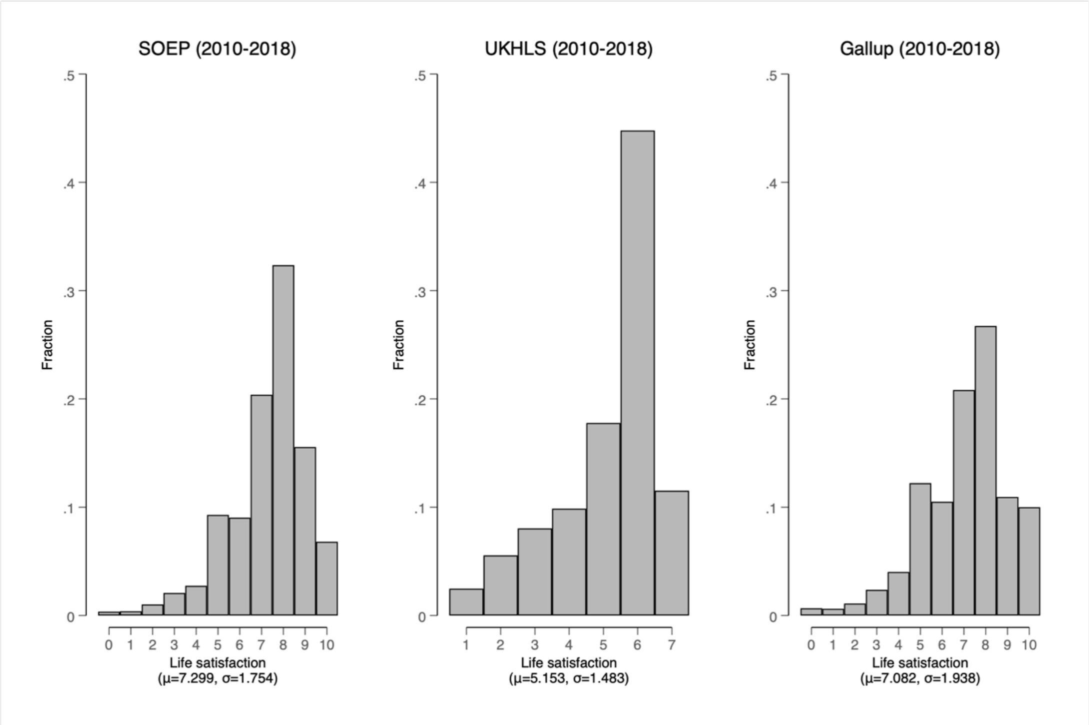

Figure A2: Differences in within-sample performance between OLS and ML. The R-squareds are calculated from the training data and are not representative of out-of-sample performance.

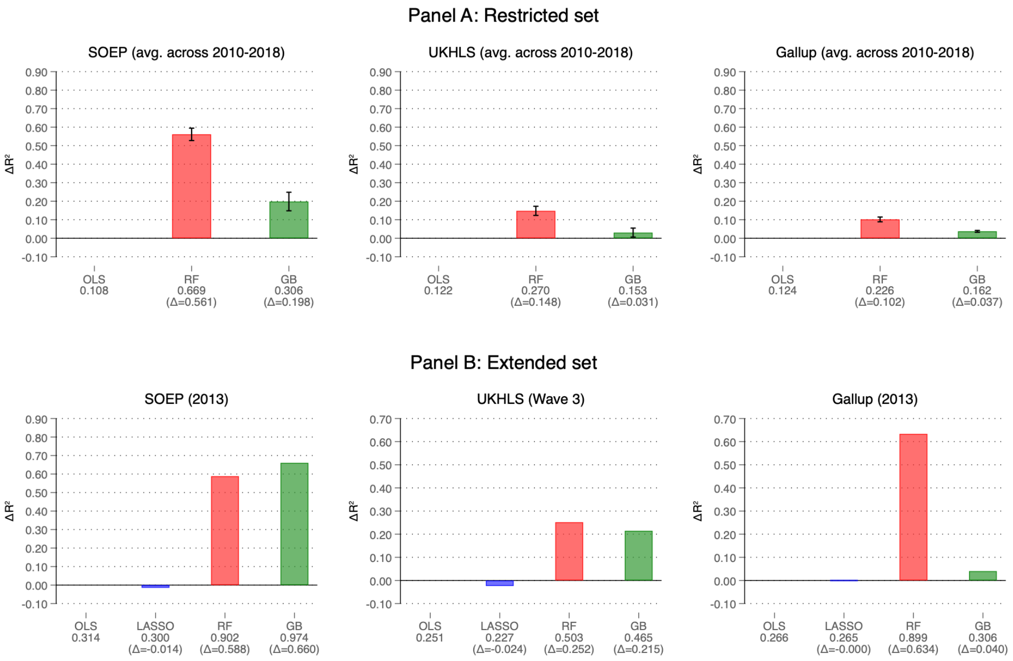

Figure A3: Relationships between wellbeing, age, and household income; conditional on the other variables in the extended set.

Panel A: SOEP

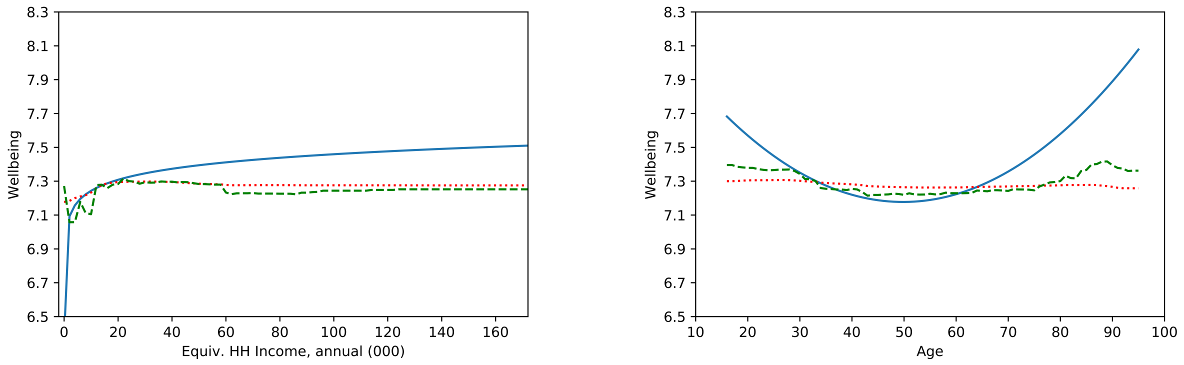

Panel B: UKHLS

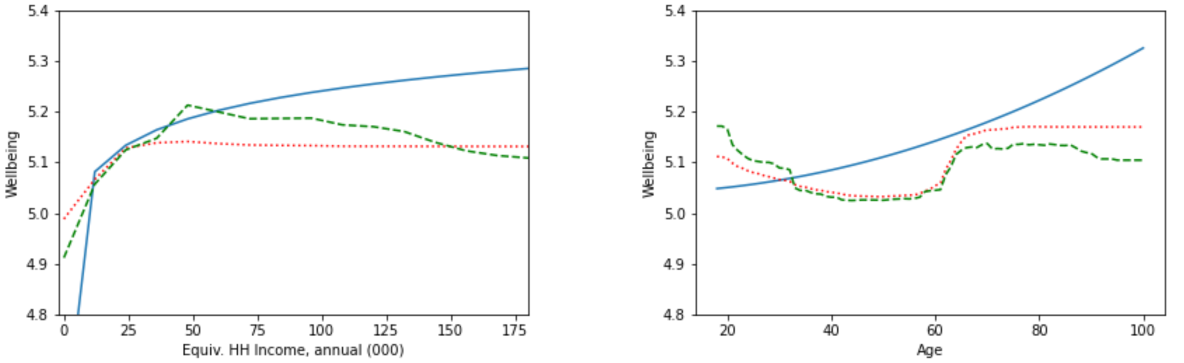

Panel C: Gallup

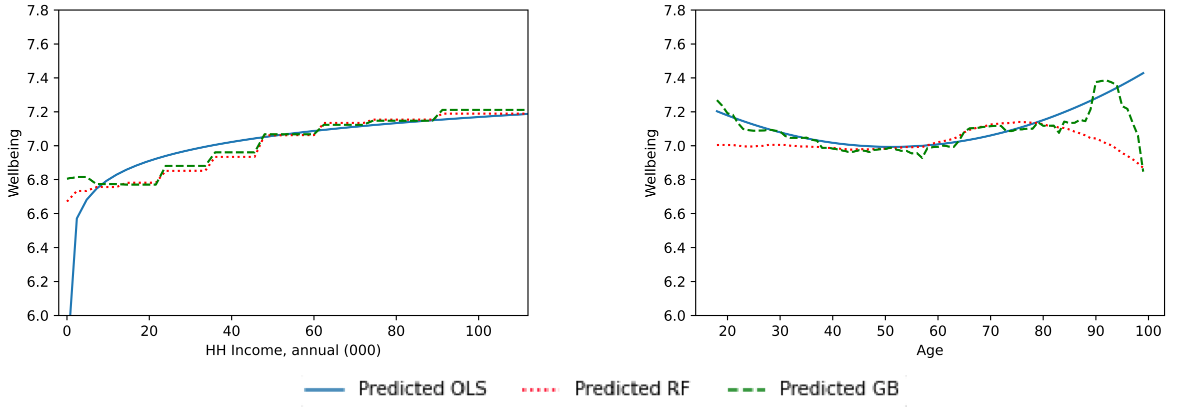

Notes: Income is continuous in the SOEP and the UKHLS, and we use equivalence-scale adjusted household income in the analysis. For ease of presentation, we only depict the relationship up to equivalent household income figures up to 180 000 in the local currency for these two datasets. Income is collected in income bands in Gallup, and there is no information on household size in 2013. The Gallup analysis thus refers to non-adjusted household income.

Figure A4: Differences in out-of-sample performance between OLS and ML when modelling positive and negative affect. Using 2013 Gallup and 2013 SOEP data with the Extended Set of variables. The R-squareds are calculated from unseen 'testing data'.

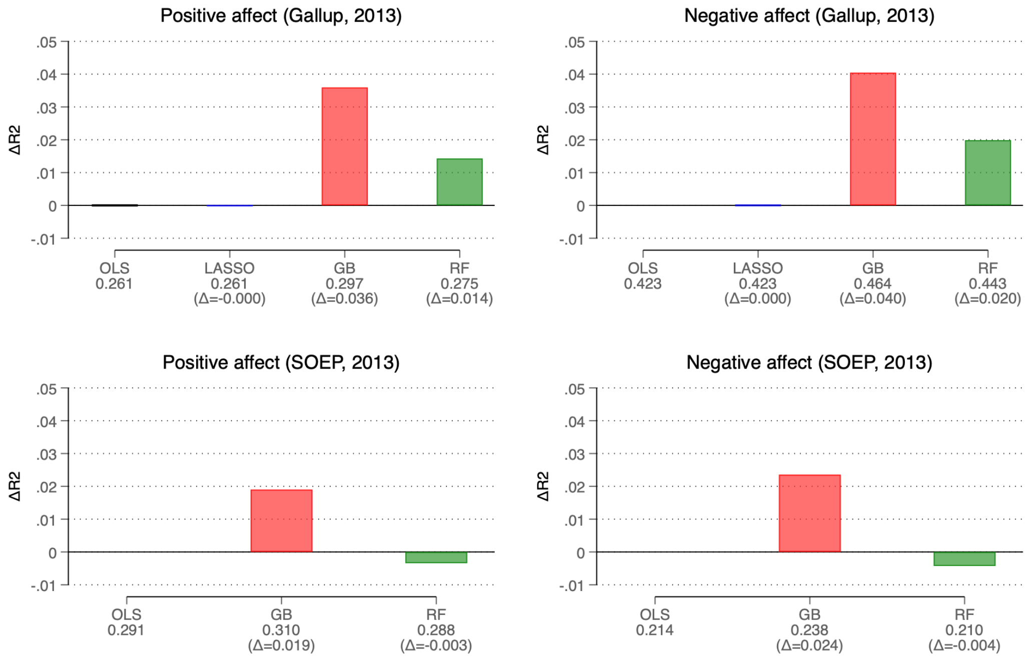

Figure A5: Differences in R-squared between OLS and when modelling the level of wellbeing with Mundlak terms using 2013 SOEP and Wave 3 UKHLS data with the Restricted Set of variables. The R-squareds are calculated from unseen 'testing data'.

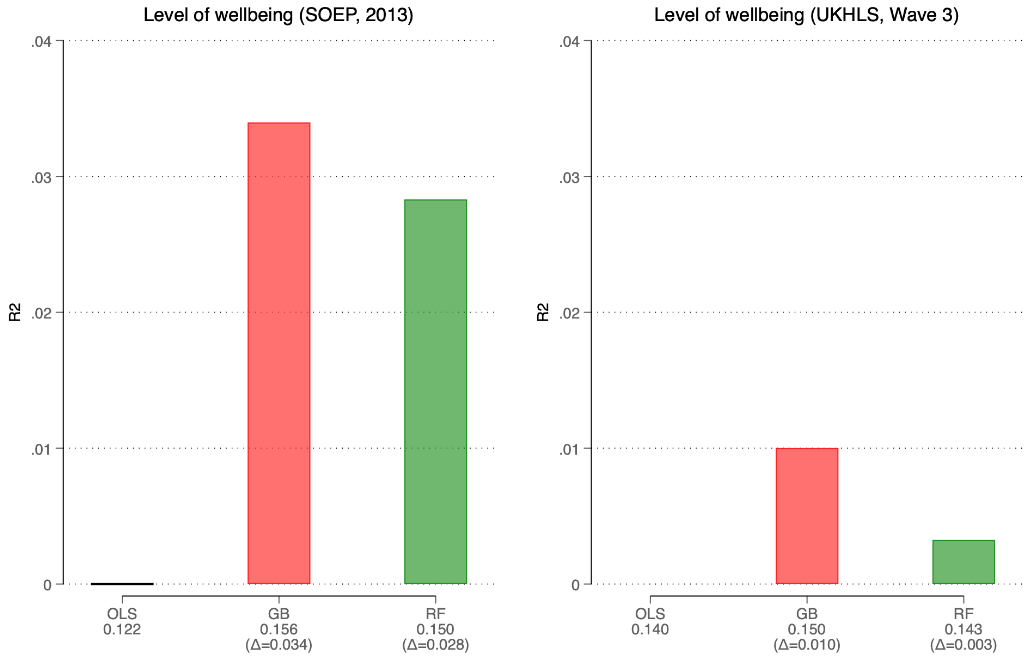

References

[1] Kahneman, D., & Deaton, A. (2010). High Income Improves Evaluation of Life but not Emotional Well-Being. *PNAS*, *107*(38), 16489–93.

[2] Mundlak, Y. (1978). On the Pooling of Time Series and Cross Section Data. *Econometrica*, *46*(1), 69–85.

[3] Wooldridge, J. M. (2010). *Econometric Analysis of Cross-Section and Panel Data*. MIT press.

[4] Hastie, T., Tibshirani, R., Friedman, J. H., & Friedman, J. H. (2009). *The Elements of Statistical Learning: Data Mining, Inference, and Prediction* (Vol. 2). Springer.

[5] Chen, T., & Guestrin, C. (2016). Xgboost: A Scalable Tree Boosting System. *Proceedings of the 22nd ACM SIGKDD international conference on knowledge discovery and data mining*, 785–794.
